# ^18^F-FDG PET and PET/CT for the evaluation of gastric signet ring cell carcinoma: a systematic review

**DOI:** 10.1097/MNM.0000000000001481

**Published:** 2021-08-26

**Authors:** Francesco Dondi, Domenico Albano, Raffaele Giubbini, Francesco Bertagna

**Affiliations:** Nuclear Medicine, University of Brescia and ASST Spedali Civili di Brescia, Brescia, Italy

**Keywords:** 18F-fluorodeoxyglucose, gastric cancer, gastric signet ring cell cancer, PET, SRCC

## Abstract

In the last years, ^18^F-fluorodeoxyglucose PET/computed tomography (^18^F-FDG PET/CT) has demonstrated its utility for the evaluation of gastric cancer; however, considering some histotypes such as gastric signet ring cell carcinoma (GSRCC) the results are limited. The aim of this review is to analyze the diagnostic performance of ^18^F-FDG PET and PET/CT for the assessment of GSRCC.

**Methods:**

A wide literature search of the *PubMed*/*MEDLINE*, *Scopus*, *Embase* and *Cochrane library* databases was made to find relevant published articles about the diagnostic performance of ^18^F-FDG PET or PET/CT for the evaluation of GSRCC.

**Results:**

The comprehensive computer literature search revealed 179 articles. On reviewing the titles and abstracts, 162 articles were excluded because the reported data were not within the field of interest. Nine studies were included in the review and references were also screened for additional articles. Finally, 26 articles were selected and retrieved in full-text version.

**Conclusion:**

Despite some limitations affect our review, GSRCC seems to have low ^18^F-FDG uptake, and therefore ^18^F-FDG PET or PET/CT reveals impaired sensitivity for its evaluation. However, a correlation between ^18^F-FDG uptake and some clinico-pathologic features (such as stage, depth of invasion, size and presence of nodal metastasis) has been demonstrated. Besides, a possible prognostic role of PET/CT features is starting to emerge.

## Introduction

Gastric cancer is one of the most common and aggressive cancer worldwide. Despite the decrease in incidence in the last decades, the survival rate remains low because the clinical presentation is often absent or nonspecific [1,2].

Gastric cancer can be histologically divided according to a different classification. World Health Organization (WHO) is one of the most used such as Lauren classification that divides gastric cancer mainly into intestinal and diffuse cancer, comprising gastric signet ring cell cancer (GSRCC) and other types [3].

Complete surgical removal of the primitive tumor with nodal dissection is a pivotal part of the treatment of disease. However, recurrence is often present, ranging from 22 to 48% of the cases [4].

Accurate and early staging of the disease is fundamental for treatment planning and nowadays a complete workup includes computed tomography (CT), gastroscopy and laparoscopy [1]. In the last years, ^18^F-fluorodeoxyglucose PET/computed tomography (^18^F-FDG PET/CT) has increased its role for staging and restaging of disease, because of its ability to provide both anatomic and functional information. Its use has been widely demonstrated especially in treatment planning [5].

Stomach evaluation with ^18^F-FDG PET/CT may be challenging due to the risk of having heterogeneous gastric uptake distribution owing to different inflammatory and physiologic states. Besides, diabetic patients, especially in therapy with oral hypoglycemic drugs, may present an increased tracer uptake in the bowel, which affects and limits the evaluation of the scan in the gastrointestinal tract [6,7].

Despite the proven usefulness of ^18^F-FDG PET/CT for the evaluation of patients affected by gastric cancer, its role remains controversial given its low general sensitivity [8]. In particular, gastric cancer seems to be non ^18^F-FDG-avid in a high percentage of cases, up to 53% as reported in the literature, and impaired ^18^F-FDG uptake is particularly evident when GSRCC is present [1]. The aim of this systematic review is to analyze the performance of ^18^F-FDG PET or PET/CT for the assessment of GSRCC.

## Materials and methods

### Search strategy

A wide literature search of the *PubMed*/*MEDLINE*, *Scopus*, *Embase* and *Cochrane library* databases was made to find significant published articles concerning the usefulness of PET and PET/CT for the evaluation of GSRCC. Search algorithms were the following: (1) “signet ring cell” AND “PET”, (2) “SRCC” and “PET”, (3) “signet” AND “ring” AND “cell” AND “PET”, (4) “signet ring cell” AND “PET”, (5) “SRCC” and “PET” and (6) “signet” AND “ring” AND “cell” AND “positron” AND “emission” AND “tomography”.

No beginning date limit was applied to the search and it was updated until 31 October 2020. Only articles in the English language were considered; preclinical studies, conference proceedings, reviews and editorials were excluded. To expand our search, the references of the retrieved articles were also screened for additional papers.

### Study selection

Two researchers (F.D. and D.A.) independently reviewed the titles and the abstracts of the retrieved articles. The same two researchers then independently reviewed the full-text version of the remaining articles to determine their eligibility for inclusion. The studies with less than eight patients affected by GSRCC were conventionally excluded from the review due to the low sample analyzed. The quality assessment, including the risk of bias and applicability concerns, was carried out using Quality Assessment of Diagnostic Accuracy Studies (QUADAS)-2 evaluation [9].

### Data abstraction

For each included study, data concerning the basic study were collected (author names, year of publication, country of origin and type of study) and PET device used, number of patients evaluated and number of patients affected by GSRCC. The main findings of the articles included in this review are reported in the Results.

## Results

### Literature search

**Fig. 1:**
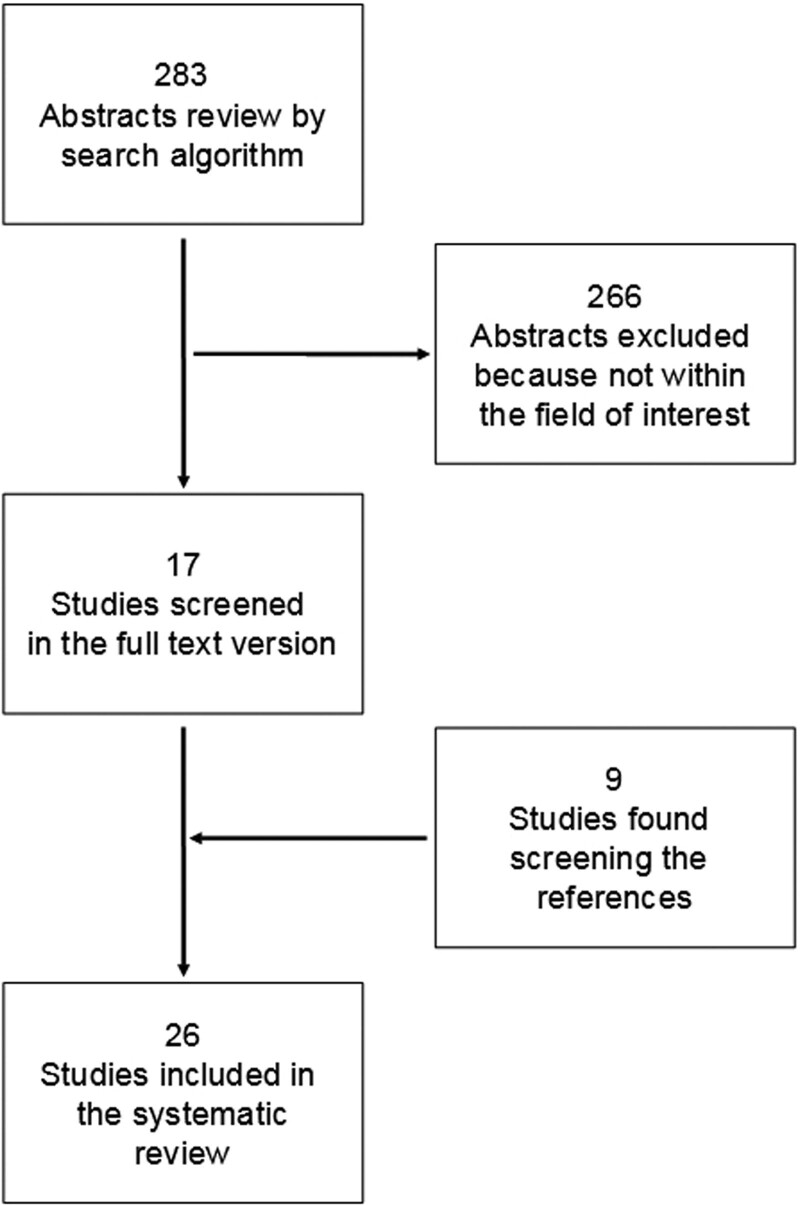
Flow chart of the search of eligible studies on the diagnostic performance of ^18^F-FDG PET or PET/CT for the assessment of GSRCC.

A total of 283 articles were extrapolated with the computer literature search; by reviewing the titles and abstracts, 266 of them were excluded because the reported data were not within the field of interest of this review. Seventeen articles were selected and retrieved in full-text version [10–26]; nine additional studies were also found screening the references of these articles [27–35]. A total of 26 articles were then included in the systematic review [10–35] (Fig. 1).

**Fig. 2:**
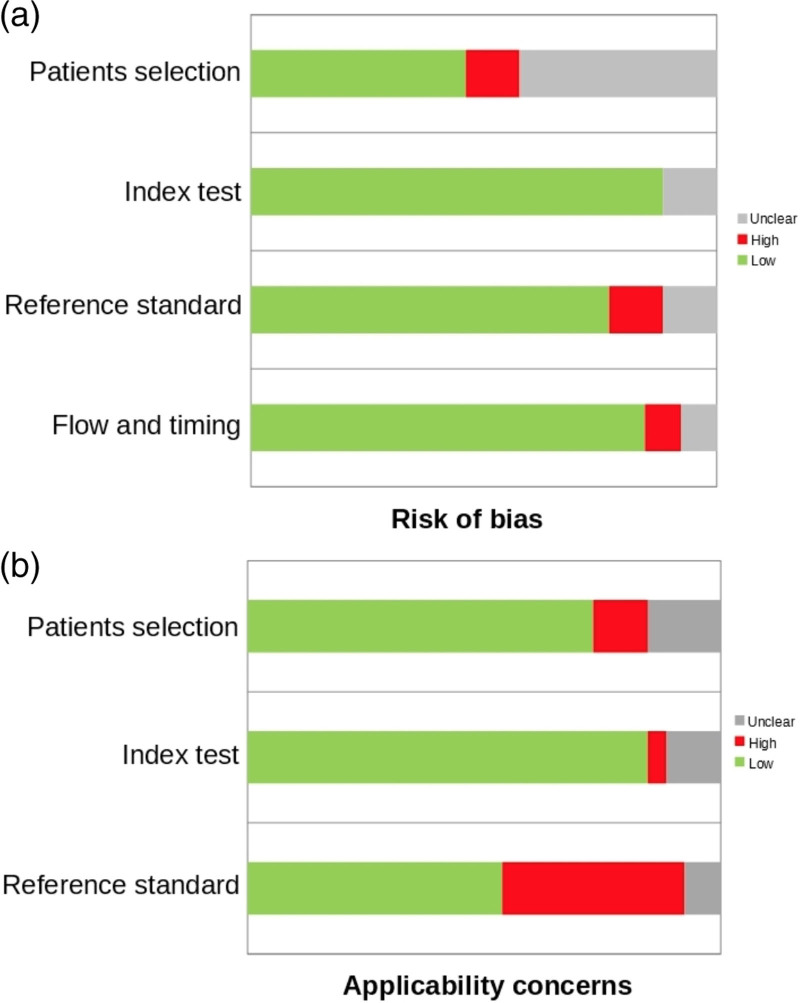
QUADAS-2 quality assessment for risk of bias (2a) and applicability concerns (2b). QUADAS, Quality Assessment of Diagnostic Accuracy Studies.

In general, the quality assessment using QUADAS-2 evaluation underlined a low risk of bias (Fig. 2a) and low problem regarding applicability concerns (Fig. 2b).

Among the 26 studies included in the systematic review, 24 were of retrospective nature [10–17,19–31,33–35] while two were prospective one [18,32]. In 9 studies the device used was PET only [10,11,13–15,17,27,28,30], in 1 study both PET and PET/CT were used [18], while in the remaining 16 studies hybrid PET/CT tomograph was used [12,16,19–26,29,31–35]. ^18^F-FDG was used in all the considered studies, except for one work in which both ^18^F-FDG and ^18^F-fluorothymidine (^18^F-FLT) were used [18]. The main characteristics of the studies and their results are briefly presented in Tables 1 and 2.

**Table 1:** Characteristics of the studies considered for the review

First author	References	Year	Country	Study Design	Isotope	N. Pts	Gender male:Female	GSRCC pts
De Potter *et al*.	10	2002	Belgium	Retrospective	^18^F-FDG	20	16:4	8 (40.0%)
Yoshioka *et al*.	11	2003	Japan	Retrospective	^18^F-FDG	42	29:13	8 (19.0%)
Stahl *et al*.	28	2003	Germany	Retrospective	^18^F-FDG	40	27:13	11 (27.5%)
Mochiki *et al*.	27	2004	Japan	Retrospective	^18^F-FDG	85	55:30	9 (10.6%)
Chen *et al*.	15	2005	South Korea	Retrospective	^18^F-FDG	73	61:12	9 (12.3%)
Kim *et al*.	14	2006	Korea	Retrospective	^18^F-FDG	68	49:19	12 (18%)^a^
Yamada *et al*.	13	2006	Japan	Retrospective	^18^F-FDG	35	24:11	9 (25.7%)
Herrmann *et al*.	18	2007	Germany	Prospective	^18^F-FDG, ^18^F-FLT	45	31:14	27 (60%)
Park *et al*.	34	2009	Korea	Retrospective	^18^F-FDG	105	75:30	23 (21.9%)
Hur *et al*.	33	2010	Korea	Retrospective	^18^F-FDG	133	92:41	25 (18.8%)^a^
Alakus *et al*.	17	2010	Germany	Retrospective	^18^F-FDG	35	28:7	17 (48.6%)
Choi *et al*.	19	2010	Korea	Retrospective	^18^F-FDG	40	28:12	40 (100%)
Kim *et al*.	35	2011	Korea	Retrospective	^18^F-FDG	136	88:51	19 (14.0%)
Ha *et al*.	12	2011	Korea	Retrospective	^18^F-FDG	78	53:25	17 (21.8%)
Pak *et al*.	16	2011	Korea	Retrospective	^18^F-FDG	41	19:22	41 (100.0%)
Youn *et al*.	31	2012	Korea	Retrospective	^18^F-FDG	396	278:118	92 (23.2%)
Smyth *et al*.	32	2012	USA	Prospective	^18^F-FDG	113	68:45	52 (46.0%)^c^
Lee *et al*.	20	2012	Korea	Retrospective	^18^F-FDG	271	171:100	31 (11.4%)^a^
Takebayashi *et al*.	30	2013	Japan	Retrospective	^18^F-FDG	50	29:21	25 (50.0%)^b^
Park *et al*.	21	2014	Korea	Retrospective	^18^F-FDG	74	56:18	16 (22%)
Charalampakis *et al*.	23	2015	USA	Retrospective	^18^F-FDG	60	38:22	31 (51.6%)
Chen *et al*.	25	2016	China	Retrospective	^18^F-FDG	64	38:26	19 (29.7%)
Park *et al*.	29	2018	Korea	Retrospective	^18^F-FDG	124	80:44	28 (22.6%)
Chon *et al*.	22	2019	Korea	Retrospective	^18^F-FDG	727	494:233	110 (15.1%)
Harada *et al*.	24	2020	USA	Retrospective	^18^F-FDG	59	43:16	26 (44.1%)
Arslan *et al*.	26	2020	Turkey	Retrospective	^18^F-FDG	341	256:85	92 (26.9%)

; ^18^F-FDG, ^18^F-fluorodeoxyglucose; ^18^F-FLT, ^18^F- fluorothymidine; GSRCC, gastric signet ring cell carcinoma; Pts, patients.

a GSRCC and mucinous cancer.

b Nonintestinal cancers.

c Diffuse histology cancer.

**Table 2:** Results and main findings of the studies considered for the review

First author	Device	Activity mean (MBq)	Uptake time (min)	PET analysis	Setting	GSRCC PET positive	Mean SUV	Main findings
De Potter *et al*.	PET	6.5 MBq/kg	60	Qualitative	Restaging	5 (62.5%)	NS	Detection rate for GSRCC is lower than non-GSRCC
Yoshioka *et al*.	PET	222 ± 72	30–45	Qualitative and semiquantitative	Staging and restaging	NS	7.7 ± 2.6	Lower SUV for GSRCC and nodal metastasis^a^
Stahl *et al*.	PET	300	40	Qualitative and semiquantitative	Staging, pretreatment	NS	4.8 ± 2.8^b^	Lower detection rate and SUV for nonintestinal cancers.
Mochiki *et al*.	PET	275–370	40	Qualitative and semiquantitative	Staging, preoperative	7 (77.8%)	NS	Detection rate for GSRCC is similar to non-GSRCC
Chen *et al*.	PET	370–555	60	Qualitative, 3-point scale and semiquantitative	Staging, preoperative	NS	4.2	Lower SUV_max_ for GSRCC and mucinous cancer
Kim *et al*.	PET	555	60	Qualitative, 5-point scale and semiquantitative	Staging, preoperative	8 (88.9%)	4.7 ± 1.7	Lower sensitivity and SUV_max_ for nodal GSRCC metastasis
Yamada *et al*.	PET	200–300	50–60	Qualitative and 4-point scale	Staging, preoperative	6 (66.6%)	NS	Detection rate for GSRCC is lower than non-GSRCC. Low GLUT-1 expression for GSRCC^c^
Herrmann *et al*.	PET and PET/CT	300–370 MBq FGD, 300 MBq FLT	60 for FDG, after injection for FLT	Qualitative and semiquantitative	Staging, preoperative	27 (100%) for ^18^F -FLT 16 (59.3%) for ^18^F -FDG	5.4 for ^18^F-FLT, 6.4 for ^18^F-FDG	Higher sensitivity for ^18^F-FLT than ^18^F-FDG, but lower ^18^F-FLT and FDG uptake for GSRCC than non-GSRCC
Park *et al*.	PET/CT	370	45	Qualitative and semiquantitative	Restaging	NS	NS	Higher rate of false negative for GSRCC
Hur *et al*.	PET/CT	440	60	Qualitative and semiquantitative	Staging, preoperative	22 (88.0%)^d^	NS	No differences in detection rate for primary and nodal metastasis between GSRCC and non-GSRCC
Alakus *et al*.	PET	370	60	Qualitative and semiquantitative	Staging, preoperative	NS	3.0	Lower SUV_max_ for GSRCC. Correlation between SUV_max_ and GLUT-1 expression
Choi *et al*.	PET/CT	370	60	Qualitative and semiquantitative	Staging, preoperative	17 (42.5%)	NS	Correlation between SUV_max_ and some clinicopathological features
Kim *et al*.	PET/CT	296–444	55–60	Qualitative and semiquantitative	Restaging	NS	NS	No different accuracy in detecting recurrence between GSRCC and non-GSRCC
Ha *et al*.	PET/CT	5–6 MBq/kg	60	Qualitative and semiquantitative	Staging, preoperative	6 (35.3 %)	NS	Detection rate for primary and nodal localization in GSRCC is lower than non-GSRCC
Pak KH	PET/CT	30–444	60	Qualitative and semiquantitative	Staging, preoperative	NS	3.8	Correlation between some clinicopathological features and SUV_max_. Worse survival for higher SUV_max_
Youn *et al*.	PET/CT	NS	60	Qualitative	Staging, preoperative	29 (31.5%)	NS	Lower detection rate for SRCC and diffuse carcinomas
Smyth *et al*.	PET/CT	NS	65 ± 10	Qualitative and 5-point scale	Staging, preoperative	23 (44.2%)^e^	NS	Lower detection rate for diffuse carcinomas and low utility for metastatic lesions
Lee *et al*.	PET/CT	5.18 MBq/kg	60	Qualitative and semiquantitative	Staging, prevision of recurrence	12 (38.7%)	7.4 ± 9.3	Lower sensitivity for GSRCC than non-GSRCC. Marginal correlation between ^18^F-FDG uptake and prognosis in GSRCC.
Takebayashi *et al*.	PET	NS	NS	Qualitative and semiquantitative	Staging, preoperative	NS	9.2 ± 1.0^b^	Nonintenstinal gastric cancer show higher SUV_max_ and GLUT-1 expression
Park *et al*.	PET/CT	7.4 MBq/kg	60	Qualitative and semiquantitative	Staging, preoperative	NS	NS	Similar sensitivity for PET/CT and CECT in GSRCC for primary tumor and nodal metastasis assessment, in contrast with non-GSRCC
Charalampakis *et al*.	PET/CT	NS	NS	Qualitative and semiquantitative	Staging, previous to therapy	NS	NS	Lower SUV for GSRCC and tendency for shorter OS.
Chen *et al*.	PET/CT	3.7 MBq/kg	50 ± 6	Qualitative and semiquantitative	Staging, preoperative	NS	3.2 ± 1.2	GSRCC has lower SUV_max_ and lower HER2 expression than non-GSRCC.
Park *et al*.	PET/CT	5.5 MBq/kg	60	Qualitative and semiquantitative	Restaging	NS	NS	GSRCC has low HER2 expression and lower semiquantitative parameters.
Chon *et al*.	PET/CT	5.5 MBq/kg	60	Qualitative and semiquantitative	Staging, preoperative	75 (68.2%)	4.5 ± 1.9	Lower sensitivity and SUV_max_ for GSRCC and diffuse cancer. Weak correlation between SUV_max_ and size. High SUV_max_ is a poor prognostic factor.
Harada *et al*.	PET/CT	333–629	60–90	Qualitative and semiquantitative	Staging, prechemoradiation	NS	5.8	Lower SUV_max_ and TLG for GSRCC. Absence of GSRCC is associated with response to therapy.
Arslan *et al*.	PET/CT	3.7–5.2 MBq/kg	60	Qualitative and semiquantitative	Staging, preoperative	90 (97.8%)	9.7 ± 7.6	GSRCC has lower SUV_max_. Higher SUV_max_ for primary GSRCC in presence of nodal and distant metastasis

^18^F-FDG, ^18^F-fluorodeoxyglucose; ^18^F-FLT, ^18^F- fluorothymidine; CECT, contrast-enhanced computed tomography; GLUT-1, glucose transporter 1; GSRCC, gastric signet ring cell carcinoma; HER2, human epidermal growth factor receptor 2; kg, kilograms; MBq, megabecquerel; NS, not specified; OS, overall survival; Pts, patients; SUV, standardized uptake value; TLG, total lesion glycolysis.;

a GSRCC and poorly differentiated carcinoma.

b Nonintestinal histology.

c GSRCC, nonsolid carcinoma and poorly differentiated carcinoma.

d SRCC and mucinous cancer.

e Diffuse histology.

### ^18^F-FDG avidity and diagnostic accuracy of PET and PET/CT

Several studies have reported that ^18^F-FDG PET and PET/CT imaging has impaired accuracy for the evaluation of GSRCC, compared to other gastric cancer histotypes.

In particular, the sensitivity and specificity of ^18^F-FDG imaging are higher for non-GSRCC compared to GSRCC [10,12,18,22,31]. Furthermore, a higher rate of false-negative scans is described in patients affected by GSRCC [10,14,26,34]. In general, the criteria used to define PET scan positivity or negativity were comparable among the studies considered in the review and consisted of visual and semiquantitative evaluation for most of the studies. PET scan was considered positive when an increased FDG uptake higher than background activity in the surrounding tissue and blood pool was present; positron imaging was also compared to other imaging modalities or hystopathologic evidence when available.

Regarding diagnostic accuracy of ^18^F-FDG PET/CT for the evaluation of GSRCC, the data included in the review are very heterogeneous. The detection rate of PET/CT ranges from 14 to 98% of the cases, depending on hystopatologic classification used in each study. Furthermore, the sensitivity varies from 15 to 63% and the specificity from 60 to 100% considering only GSRCC, but only small amounts of the studies clearly report these informations.

Moreover, the lower ^18^F-FDG uptake of GSRCC has been underlined by many studies and standardized uptake value (SUV) is usually lower compared to other gastric cancer subtypes [10,11,18,22,25,28]. Significant differences in terms of the detection rate and SUV values between intestinal and nonintestinal gastric cancer have also been reported such as a strong correlation between the presence of mucus and impaired ^18^F-FDG uptake, with the presence of higher SUV in nonmucus-containing tumors [15,20,28]. These findings are also true when comparing GRSCC and poorly-differentiated gastric cancer to well-differentiated forms [11,32]. Moreover, diffuse gastric cancer presented low positivity of primary lesions compared to other histotypes [32]. Interestingly, some clinicopathologic parameters such as stage, depth of invasion, size, presence of nodal metastasis and lymphovascular invasion are demonstrated to be correlated with the degree of ^18^F-FDG uptake [16]. Despite the similarity of SUV between different gastric cancer histotypes, diffuse-type tumors tend to have larger primary tumor than other types [14]. Higher _SUVmax_, SUV_mean_, total lesion glycolysis (TLG) and metabolic tumor volume (MTV) have also been reported for well-differentiated and moderately-differentiated cancer compared to poorly-differentiated and GSRCC cancer [29].

Otherwise, some studies report no differences in terms of ^18^F-FDG uptake and sensitivity between GSRCC and non-GSRCC cancer and surprisingly in some cases GSRCC demonstrates higher SUV [27,30,33,35].

As primary lesions, lymph node localization of GSRCC tends to be less ^18^F-FDG avid than other gastric cancer nodal metastasis and therefore PET imaging has impaired sensitivity [11,14,21,26].

### Correlation of ^18^F-FDG uptake and glucose transporter-1 or human epidermal growth factor receptor 2 expression

Correlation between ^18^F-FDG uptake and glucose transporter-1 (GLUT-1) expression in gastric cancer has been reported. In particular, GSRCC has significantly lower expression of this transporter than other gastric cancer histotypes, resulting in impaired ^18^F-FDG uptake and detection [4,17,19].

Expression of human epidermal growth factor receptor 2 (HER2) in GSRCC has also been studied in the past. In particular, a lower HER2 expression rate in GSRCC compared to well-differentiated and moderately-differentiated cancer has been demonstrated. Moreover, this difference is not present when considering SRCC and poorly-differentiated forms of gastric cancer. [25,29]. Furthermore, PET/CT parameters such as SUV_max_, SUV_mean_, TLG and MTV are significantly higher in HER2 positive patients [29]. In contrast to what was previously reported, increased GLUT-1 expression for nonintestinal cancers has been also reported in literature [30].

### Prognostic value of ^18^F-FDG PET/CT

^18^F-FDG PET/CT is recently starting to emerge as a promising imaging method of prognostic utility in GSRCC. When considering all gastric cancer together, the presence of GSRCC or lower SUV_max_ are correlated with shorter overall survival (OS) [23,29]. However, when considering GSRCC or diffuse histology, patients with higher SUV_max_ tend to have more relapse, shorter relapse free survival, lower cancer-specific survival rate and shorter OS [16,22]. Moreover, the absence of GSRCC, higher SUV_max_ and higher TLG were associated with pathologic complete response [24]. However, these insights are not clearly supported by other findings when evaluating the role of PET/CT in predicting the recurrence of GSRCC [20].

## Discussion

Several studies have demonstrated that GSRCC generally presents lower ^18^F-FDG uptake compared to other gastric cancer histotypes; therefore, PET and PET/CT imaging has impaired accuracy for the evaluation of GSRCC. [10,12,14,18–20,22,25,26,31,32,34]. Moreover, the risk of false-negative reports is high when considering GSRCC and PD cancers [10,14,26,34]. The sensitivity and specificity of ^18^F-FDG PET or PET/CT is generally higher for non-GSRCC (75 and 75%) compared to GSRCC (63 and 60%) [10].

As previously mentioned, GSRCC is classified as nonintestinal, diffuse and mucus-containing cancer [3]. Different uptake between intestinal and nonintestinal gastric cancer have been described by Stahl *et al*., [28] despite the fact that nonintestinal tumor were higher in grade than intestinal types. Moreover, a strong correlation between the presence of mucus and impaired ^18^F-FDG uptake was also reported. Similar findings were also confirmed by other authors [14,15,18,22,23,31]. Kim *et al*., [14] reported that despite the similarity of SUV between different gastric cancer histotypes, diffuse-type tumors had significantly larger primary tumor compared to other types. Partially in contrast, in their article, Chon *et al*., [22] reported that the degree of correlation between SUV_max_ and size of primary lesion was relatively weak for patients with GSRCC or diffuse histology.

^18^F-FDG uptake, expressed as SUV_max_, may be related with some clinical and histologic features, such as depth of invasion, size, presence of nodal metastasis and lymphovascular invasion, proposed by Pak *et al*., [16]. Moreover, they reported that patients with higher SUV_max_ had larger tumors, more total gastrectomies and higher stages compared to the group of lower SUV_max_. In this context, Harada *et al*., [24] showed that high SUV_max_ and TLG were associated with the absence of GSRCC.

Nodal localizations of GSRCC, especially if regional, tends to be less ^18^F-FDG avid than other gastric tumor nodal metastasis resulting in impaired sensitivity for PET and PET/CT imaging [11,12,14,21,26]. For example, Kim *et al*., [14], underlined reduced sensitivity and SUV for GSRCC nodal metastasis and furthermore univariate analysis reported GSRCC and SUV to be significant variables for nodal metastasis detection. However, multivariate analysis confirmed only SUV as an independent predictive variable. In a recent article, Arslan *et al*., [26] demonstrated that SUV_max_ of positive regional lymph nodes were significantly lower for GSRCC. Interestingly, this difference was not confirmed when considering distant lymph nodes as also suggested by Smyth *et al*., [32] reporting that in patients with diffuse cancer, metastatic disease was more likely to be detected by laparoscopy than PET/CT.

It is known that the GLUT-1 overexpression plays a major role for ^18^F-FDG uptake by cancer cells [36]. In this context, studies about the expression of this transporter in GSRCC have been performed reporting in general reduced GLUT1 presence [13,17,19]. Yamada *et al*., [13] reported that tracer uptake and detection rate on ^18^F-FDG PET/CT differed depending on GLUT-1 expression between the GSRCC and other subtypes of gastric cancer. When applying multiple regression analysis, GLUT-1 expression was the most influential factor for predicting the degree of ^18^F-FDG uptake. Choi *et al*., [19] reported that, in GSRCC, group of membranous pattern of GLUT-1 staining had significantly higher SUV_max_ than group of cytoplasmic pattern. Significant differences were also reported in tracer uptake when comparing depth of invasion, extent of nodal metastasis and stage, where higher stage demonstrated increased SUV_max_.

Also, anti-HER2 antibodies are starting to emerge as a tool for gastric cancer treatment [37] and therefore, given the ability of HER2 cancer status to predict response to treatment, correlation between ^18^F-FDG uptake and receptor expression has been researched through the years. In general, GSRCC demonstrated lower HER2 expression compared to other gastric cancer [25,29]. In this context, Park *et al*., [29], reported that SUV_max_, SUV_mean_, TLG and MTV were significantly higher in HER2 positive patients. Interestingly, the authors also reported that in negative HER2 patients, no correlation between prognosis and PET/CT parameters was highlighted.

In the last years, general implications on the prognostic value of ^18^F-FDG PET/CT in GSRCC are starting to arise. Pak *et al*., [16] reported that for GSRCC patients with higher SUV_max_ had more relapse, shorter relapse-free survival, lower cancer-specific survival rate and lower OS. Univariate analysis demonstrated tumor nodes and metastases stage, type of gastrectomy and SUV_max_ as significant variables for the prediction of survival. More recently, Lee *et al*., [20] evaluated the possible role of ^18^F-FDG PET/CT in predicting recurrence after curative surgical resection, reporting only marginal significance was present for GRSCC when considering a negative scan as predictive of longer recurrence-free survival. The difference was significant when considering other gastric cancer subtypes. Similarly, Charalampakis *et al*., [23] underlined that patients with lower SUV and presence of GSRCC had the tendency to shorter OS. Moreover, Harada *et al*., [24] reported that in absence of GSRCC, SUV_max_ and TLG were associated with pathologic complete response. Lastly, Chon *et al*., [22] reported that in presence of diffuse or GSRCC, higher SUV_max_ was correlated to shorter disease-free survival and OS.

A singular study was proposed by Herrmann *et al*., [18] when comparing ^18^F-FDG and ^18^F-FLT PET or PET/CT for the staging of gastric cancer. ^18^F-FLT demonstrated lower uptake in GSRCC compared to other gastric cancer subtypes but higher sensitivity compared to ^18^F-FDG. The authors than suggested ^18^F-FLT as a promising tool for the evaluation of the particular aggressive histologic type of gastric cancer, such as GSRCC.

As previously mentioned, some works are partially in contrast to the findings reported. For example, Mochiki *et al*., [27] reported that histology of primary gastric cancer did not significantly influence the detection of disease on PET imaging. Similar results were obtained by Hur *et al*., [33]. Higher uptake of ^18^F-FDG by GSRCC compared to other gastric cancer was otherwise reported by Takebayashi *et al*., [30]. Surprisingly, the authors reported increased GLUT-1 expression for nonintestinal cancers. Similar results were also underlined by Kim *et al*., [35] reporting that adenocarcinoma had lower accuracy than GSRCC. In general, the low percentage of GSRCC presented in these studies is suggested by the authors as a possible explanation for these results.

### Conclusion

In conclusion, ^18^F-FDG PET or PET/CT demonstrated impaired diagnostic accuracy for the evaluation of primary GSRCC and nodal localization compared to other gastric cancer histotypes. Interestingly, the uptake and the main semiquantitative parameters (such as SUV) are generally lower for GSRCC. Reduced GLUT-1 and HER2 expression is also present in GSRCC. Lastly, a possible prognostic role of ^8^F-FDG PET/CT features is starting to emerge.

## Acknowledgements

**Conflicts of interest**

There are no conflicts of interest.
